# Flowreversal in the left internal jugular vein due to a prominent brachiocephalic trunk: A case report

**DOI:** 10.1016/j.radcr.2024.04.032

**Published:** 2024-05-03

**Authors:** Johanna Tennigkeit, Thomas Teich, Jonas Lübcke, Andreas G. Schreyer, Oliver Ritter

**Affiliations:** a Department of Cardiology, Nephrology and Pneumology, Brandenburg Medical School, Theodor Fontane, University Hospital Brandenburg/Havel, Hochstraße 29 14770, Brandenburg/Havel, Germany; b Department for Diagnostic and Interventional Radiology, Brandenburg Medical School Theodor Fontane, University Hospital Brandenburg/Havel, Hochstraße 29 14770, Brandenburg/Havel, Germany

**Keywords:** Flowreversal, Jugular vein, Brachiocephalic trunk

## Abstract

Reversal of blood flow has only been reported in the left internal jugular vein following interventions such as central venous catheter, dialysis shunt placement, or external compression from a tumor. We describe a rare case of chronic headache and hearing loss due to flow reversal in the left internal jugular vein and compensatory massive dilation of the right internal jugular vein. Flow reversal was caused by a prominent brachiocephalic trunk with subseqent compression of the vena brachiocephalica sinistra. Vascular anomalies and associated venous bypass circulation may be considered as a rare cause of non-specific malaise. Restoration of the physiological direction of blood flow should be discussed on an interdisciplinary basis given the unpredictable haemodynamic consequences.

## Graphical Abstract

**Figure:**
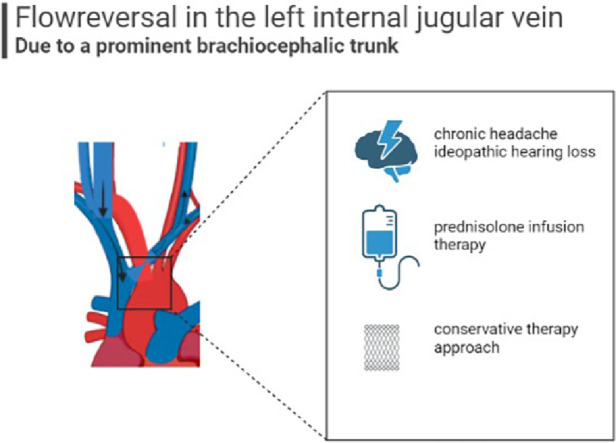

## Introduction

The brachiocephalic trunk arises as the first branch of the ascending aorta, after 2-3 cm, it divides into the right carotid artery and the right subclavian artery. In the left vein angle, the left subclavian vein, the left internal and external jugular vein unite to form the left brachiocephalic vein. The left brachiocephalic vein carries the deoxygenated blood from the left upper extremity, the left side of the head and neck region to the right heart. The left brachiocephalic vein flows ventrolateral to the brachiocephalic trunk, perpendicular to the sternum medially. It joins the right brachiocephalic vein behind the right sternoclavicular joint to form the superior vena cava. Blood flow reversal of the left internal jugular vein is observed in rare cases after vessel wall remodelling after central venous catheter or arterio-venous fistula placement. Once a diagnosis of flow reversal in a venous vessel is made, intravenous thrombosis and a vessel-compressing tumor must be excluded. We describe an uncommon case of an anatomical anomaly of the left brachiocephalic trunk causing a flow reversal in the left internal jugular vein and simultaneous excessive dilation of the right jugular vein.

## Summary figure

### Case presentation

**Fig. 1:**
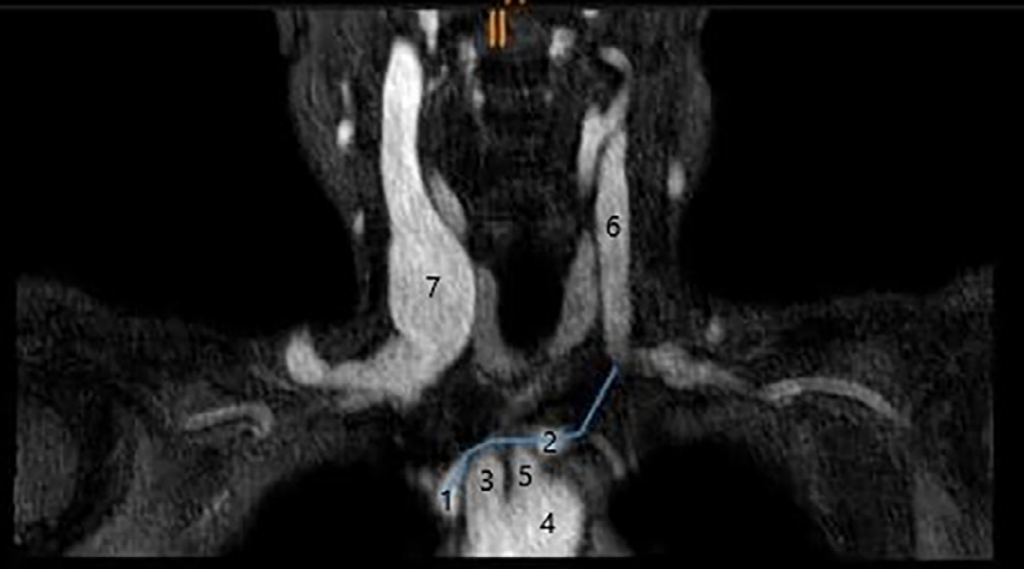
Coronal section: Excessive varicose right internal jugular vein. Contrast neck angio MRI (A,E) Tesla MRI, 4D TRAK Angiography : Vena cava superior; (B) Blue line: Vena brachiocephalica sinistra; (C) Brachiocephalic trunk; (D) Aorta descendence; (E) Arteria carotis communis sinistra; (F) Left internal jugular vein; (G) Right internal jugular vein.

**Fig. 2:**
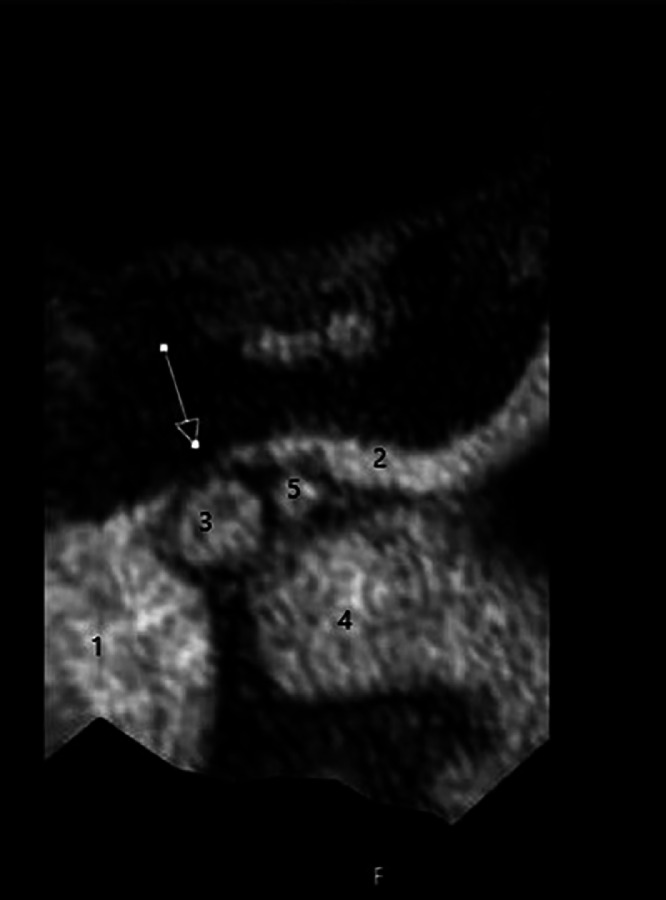
Axial section: Compression of the left brachiocephalic vein through the brachiocephalic trunk. Contrast neck angio MRI (A,E) Tesla MRI, 4D TRAK Angiography (A) Vena cava superior; (B) Vena brachiocephalica sinistra; (C) Brachiocephalic trunk; (D) Aorta descendence; (E) Arteria carotis communis sinistra; Arrow: Compression of the left brachiocephalic vein through the brachiocephalic trunk.

A 77-year-old woman was admitted to the hospital due to hearing loss in the right ear, that had been present for 1 week. The symptoms were not accompanied by dizziness or other ear noises. The patient did not remember any baro or explosion trauma. The patient presented with arterial hypertension treated with oral medication. Furthermore she described chronic headaches since childhood, which she treated occasionally with pain medication. The physical examination revealed an intact, non-irritated eardrum on both sides with ventilated tympani. A tone threshold audiometry confirmed the sensorineural hearing loss (SNHL) in the right ear. A glucocorticoid therapy was initiated according to a modified Stennert scheme. Parallel to the start of therapy, the cause of the SNHL was investigated. Blood workup showed no signs of inflammation or viral infection. Doppler sonography demonstrated normal arterial neck vessels but suspected flow reversal of the left internal jugular vein. The left basilic and brachial veins were permeable and compressible, with no internal echoes to suggest thrombosis. Overall, these findings suggested arteriovenous fistula, occlusion, or severe stenosis (intrinsic or extrinsic) of the proximal left brachiocephalic vein. A contrast neck angio MRI (1,5 Tesla MRI, 4D TRAK Angiography) was performed. Following the administration of contrast medium into the left arm, a rapid ascent of contrast was observed in the left internal jugular vein and external vein, followed by rapid outflow through the left inferior petrosal sinus, cavernous sinus, and final drainage via the right internal jugular vein. A varicose right external jugular vein (Fig. 1) were conspicuous. Initially, the left brachiocephalic vein did not fill with contrast due to a short-range constriction caused by a prominent brachiocephalic trunk (Fig. 2). Otherwise, regular contrast of the thoracic arterial vessels was subsequently seen.

A sound threshold audiometry performed on the day of discharge showed an increase of the hearing threshold between 125 and 500 Hz by 10 dB with otherwise unchanged findings compared to the admission examination. The patient herself perceived a minimal improvement in her own hearing performance after intravenous glucocorticoid therapy. An interdisciplinary team of cardiology, vascular surgery and ENT discussed the present findings. In view of the mild clinical picture, the patient's age, the patient's wishes and the risk assessment of the unforeseeable haemodynamic consequences the team decided against the establishment of physiological flow conditions. The present finding of flow reversal of the left internal jugular vein, due to a prominent brachiocephalic trunk, is considered as an incidental finding in the context of a congenital anatomical anomaly. In our opinion, there is no direct correlation to the idiopathic hearing loss. A correlation with chronic headache may be possible and cannot be ruled out.

## Discussion

This case describes a detour of venous flow with reactive dilation of the main outflow tracts. This is particularly evident in the right prominently varicose internal jugular vein, which drains the majority of blood from the the head and neck veins. The dilation of the main outflow pathway of the right internal jugular vein is caused by 2 factors: high blood volume and confluence with the right subclavian vein. The distal part of the right internal jugular vein, in particular, appears to be dilated. It seems that the confluence with the subclavian vein is not dilated or cannot dilate accordingly, resulting in dilation in front of the relative 'constriction'.

Initially, a suspicion of an atriovenous fistula in the neck vessels led to the performance of a contrast-enhanced MRI of the neck vessels. The transverse sinuses were not included in the field of view, so it cannot be definitively determined whether drainage also occurs via the lateral sinuses at the confluent sinus and the dural venous sinus. On the coronal view of the MRI images, the size of the sigmoid sinuses appears to be similar on both sides, indicating an increased outflow through the cavernous sinus.

To our knowledge, this is the first description of a most likely congenital anatomical anomaly causing blood flow reversal in an internal jugular vein. It is known that stenosis of the left brachiocephalic vein can occur after shunt insertion in haemodialysis patients [1], [2], [3] as well as after placement of a central venous catheters due to irritation of the vein wall [4,5]. External compression and thrombosis must also be excluded, but those circumstances that did not apply to this patient. In the literature, cases of brachiocephalic vein stenosis accompanied by vegetative symptoms such as arm and face swelling, temperature insensitivity [5], as well as intracranial hypertension [6], have been clinically observed. These findings were also not applicable to our patient. As the patient's headache were present since childhood, we assume a congenital fully compensated anomaly. The compression of the left brachiocephalic vein resulted in increased blood flow in the cerebral venous system. This was due to the complete venous drainage of the upper extremities, neck and head via the cerebral veins. However, we believe that the unilateral hearing loss, without further neurological abnormalities, is not directly related to the anatomical anomaly. Only favorable circumstances cannot be excluded. Generally, even after a thorough investigation, it is possible to identify a cause of SNHL in only one-third of the patients [7]. One to two-thirds of cases resolve spontaneously without intervention.

From an anatomical perspective, if the brachiocephalic vein is compressed, it is possible for outflow to occur through other collaterals such as the thyroid vein, intercostal veins, and pericardioepiphrenic anastomoses or internal thoracic collaterals, instead of the described flow reversal in the internal jugular vein. These anastomoses are typically variable and not well pronounced, and are usually small or non-existent.

Compression of veins by adjacent arteries can occur in the presence of arterial aneurysms [8]. In the literature, a respiratory-dependent compression of the left brachiocephalic vein by a prominent left subclavian artery is described, which only became symptomatic due to an artheroma in the arterial wall and the accompanying narrow mediastinum [9]. May-Thurner Syndrome, also known as iliac compression syndrome, is a condition where the left common iliac vein is compressed by the right common iliac artery due to an anatomical anomaly in the area of the iliac vessels [10].

According to Virchow's triad, changes in blood flow velocity can lead to spontaneous thrombus formation in the brachiocephalic vein. Successful attempts to cure iatrogenic vein wall changes by dilating the stenosis have been described [6]. Since the headache were described as not very troublesome and we do not see a causal correlation between the hearing loss and the altered blood flow, the interdisciplinary team decided against an intervention.

In a 3 month follow-up, the patient described a complete remission of the primary symptoms of hearing loss without further intervention or medication. Occasional headaches persisted.

## Patient consent

The authors confirm that the written consent for submission and publication of this case report, including images and associated text, has been obtained from the patient in line with COPE guidance.

## Appendix Supplementary materials

Image, video 1
